# Vaccination against Heterologous R5 Clade C SHIV: Prevention of Infection and Correlates of Protection

**DOI:** 10.1371/journal.pone.0022010

**Published:** 2011-07-20

**Authors:** Samir K. Lakhashe, Wendy Wang, Nagadenahalli B. Siddappa, Girish Hemashettar, Patricia Polacino, Shiu-Lok Hu, François Villinger, James G. Else, Francis J. Novembre, John K. Yoon, Sandra J. Lee, David C. Montefiori, Ruth M. Ruprecht, Robert A. Rasmussen

**Affiliations:** 1 Dana-Farber Cancer Institute, Boston, Massachusetts, United States of America; 2 Harvard Medical School, Boston, Massachusetts, United States of America; 3 University of Washington, Seattle, Washington, United States of America; 4 Department of Pathology and Laboratory Medicine, Emory University School of Medicine, Atlanta, Georgia, United States of America; 5 Department of Microbiology, Emory University School of Medicine, Atlanta, Georgia, United States of America; 6 Yerkes National Primate Research Center, Emory University, Atlanta, Georgia, United States of America; 7 Duke University, Durham, North Carolina, United States of America; George Mason University, United States of America

## Abstract

A safe, efficacious vaccine is required to stop the AIDS pandemic. Disappointing results from the STEP trial implied a need to include humoral anti-HIV-1 responses, a notion supported by RV144 trial data even though correlates of protection are unknown. We vaccinated rhesus macaques with recombinant simian immunodeficiency virus (SIV) Gag-Pol particles, HIV-1 Tat and trimeric clade C (HIV-C) gp160, which induced cross-neutralizing antibodies (nAbs) and robust cellular immune responses. After five low-dose mucosal challenges with a simian-human immunodeficiency virus (SHIV) that encoded a heterologous R5 HIV-C envelope (22.1% divergence from the gp160 immunogen), 94% of controls became viremic, whereas one third of vaccinees remained virus-free. Upon high-dose SHIV rechallenge, all controls became infected, whereas some vaccinees remained aviremic. Peak viremia was inversely correlated with both cellular immunity (p<0.001) and cross-nAb titers (p<0.001). These data simultaneously linked cellular as well as humoral immune responses with the degree of protection for the first time.

## Introduction

According to recent UNAIDS estimates (www.UNAIDS.org), approximately 34 million people are infected with HIV; of these, ∼56% harbor HIV-1 clade C (HIV-C). Extensive efforts have focused on developing AIDS vaccines. Disappointing results from the STEP trial, which sought to induce cellular immunity, point to the need to induce balanced virus-specific immunity, including humoral responses [1], [2]. Data from the recent RV144 Phase III Thai trial support this concept and showed that separate immunogens, each designed to individually induce either antibody-based or cell-based HIV-specific immunity, conferred some protection (31.2%) when used in combination [3].

Due to the lack of natural protective immunity against HIV, correlates of protection can be defined only in the context of at least partially effective vaccines [4]. Defining correlates of protection would allow rational improvements in candidate vaccines and immunization protocols. Since RV144 is the only HIV-1 vaccine trial that has shown partial efficacy, extensive efforts are underway to define correlates of protection using samples collected during this trial [5]; to date, however, no definite correlates have been identified. In this context, information gained from biologically relevant animal models where vaccinees have resisted immunodeficiency virus challenges can serve as a source of information to define potential correlates of protection against HIV-1.

Simian immunodeficiency virus (SIV)-infected rhesus macaques (RM) develop a disease spectrum similar to HIV-infected humans, and consequently, the SIV/RM model has been used to test the efficacy of AIDS vaccine candidates. However, SIV Env differs substantially from HIV-1 Env and thus, recombinant simian-human immunodeficiency viruses (SHIVs) have been derived that encode HIV-1 envelopes, the target of neutralizing antibody (nAb) responses. SHIVs thus allow assessing the protective role of anti-HIV-1 nAb responses in vivo. The first-generation of SHIV strains, however, reflected neither the biology of transmitted HIV-1 nor the early events in host infection observed during acute HIV-1 infection in humans. One of the earlier SHIVs, SHIV89.6P, encodes a dual-tropic primary HIV-1 envelope that exhibits CXCR4 tropism in vivo and irreversibly destroys the CD4 T-cell population within 2 weeks; most RM fail to seroconvert due to this acute pathogenicity [6]. The latter was linked to the preference of SHIV89.6P for naïve T cells [7]. In contrast, acutely transmitted HIV-1 almost always has exclusive CCR5 coreceptor usage and targets the memory CD4 T-cell population. Of note, R5 tropism is observed in almost all HIV-1 transmission events linked to mucosal exposure, which is the dominant mode (∼90%) of all HIV-1 acquisitions (reviewed in [8]). Ideally, a biologically relevant SHIV challenge strain used to assess the efficacy of candidate AIDS vaccines should reflect the nature of newly acquired HIV-1 in humans, including R5 tropism and gradual disease progression [9].

In order to develop biologically relevant challenge viruses, we constructed a series of novel SHIVs [10], [11], [12] that are exclusively CCR5 tropic and cause gradual disease progression [13], [14]. These SHIVs encode HIV-C envelopes and offer the advantage of reflecting the world's most prevalent HIV-1 subtype. One of these constructs, SHIV-1157ipEL-p [12] encodes *env* of HIV1157i, an R5 HIV-C isolated from a relatively recently infected Zambian infant. The SHIV backbone was derived from SHIV-1157ipd3N4 [10], originally built from SIVmac239 but engineered to contain additional NF-κB sites in the long-terminal repeats (LTRs) to boost virus replication. HIV-C strains generally have additional NF-κB sites in their LTRs [15] and thus, SHIV-1157ipd3N4 and SHIVs based upon its backbone represent this unique HIV-C feature. Both SHIV-1157ipd3N4 and SHIV-1157ip (a variant containing the same *env* as SHIV-1157ipEL-p) have caused AIDS in RM [13], [14]. Considering these features, i.e., exclusive R5 tropism of an envelope derived from a recently acquired HIV-C plus the fact the both components used to build the new SHIV-1157ipEL-p chimera had pathogenic potential, we used the latter as challenge virus.

To better reflect the biology of HIV-1 transmission among humans, we modified the virus challenge regimen; instead of challenging vaccinated RM with a single, high-dose of virus, we used five weekly lower-dose intrarectal (i.r.) SHIV challenges in an attempt to approximate the relatively low inocula thought to be involved in mucosal HIV-1 exposures. Importantly, primate challenge studies should employ viruses that are heterologous to those used for vaccine preparation since human vaccinees are unlikely to be exposed to HIV-1 strains exactly matching the vaccines [9]. To fulfill these conditions, we mismatched the HIV-C Env immunogen with the envelope carried by SHIV-1157ipEL-p.

Several viral and bacterial vectors that encode HIV-1 proteins have been tested in animal studies as well as in human clinical trials. The latter have shown that preexisting anti-vector immunity is a major concern regarding the immunogenicity of novel vaccine vectors [16], [17]. Some vectors may potentially enhance host susceptibility to HIV-1 infection via immune activation, e.g., homing of activated CD4^+^ T cells to mucosal sites [18], or activation of dendritic cell (DC)/T-cell interactions [19]. The potential of integration into the host cell genome is also a concern for some viral vectors.

We explored a recombinant protein-only immunization approach to bypass such concerns. This would induce strong humoral and CD4^+^ T-cell responses; CD8^+^ T-cell responses can also be expected due to antigen cross-presentation. To note, a human DC subset that efficiently cross-presents soluble proteins (equivalent to mouse CD8^+^ DC) has been recently reported by independent investigators (reviewed in [20]). Macaques were immunized with SIV Gag-Pol particles derived from SIVmac239 or SIVmne (homologous or partially heterologous to the challenge SHIV, respectively), multimeric HIV-C gp160 and HIV-1 Tat. Importantly, the gp160 immunogen was derived from HIV1084i [21], a recently transmitted HIV-C with an *env* gene that was phylogenetically distinct from SHIV-1157ipEL-p *env*. Vaccination induced cellular as well as humoral immunity – at high levels in some vaccinees, which were completely protected from viremia. We found a highly significant correlation between the responses induced by both arms of the immune system and the degree of protection.

## Results

### Vaccine safety and immunogenicity

**Figure 1:**
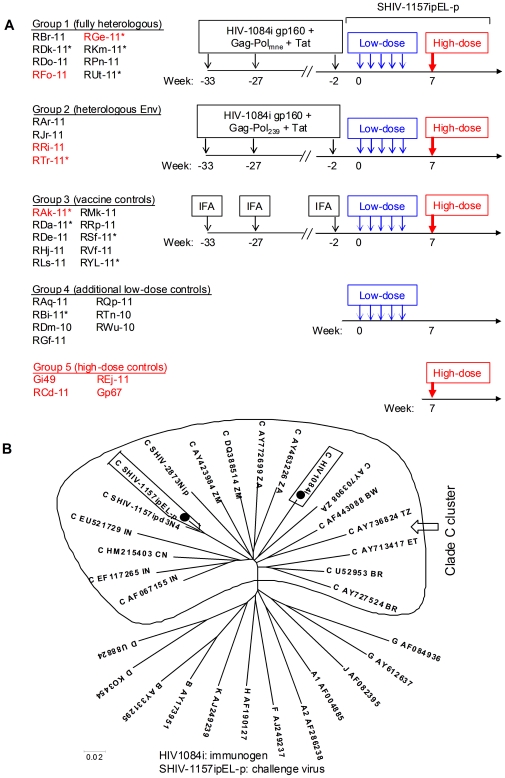
Experimental time line and phylogenetic analysis of Env of immunogen and challenge virus. (A) Experimental design. Groups 1 and 2 were vaccinated with HIV-1 Tat, HIV1084i gp160, and Gag-Pol particles from either SIVmne (Group 1) or SIVmac239 (Group 2; 100 µg of each immunogen i.m. in incomplete Freund's adjuvant (IFA)); Group 3 controls received IFA alone. Maximally 5 weekly low-dose i.r. challenges of SHIV-1157ipEL-p were given (8,000 50% tissue culture infectious doses (TCID_50,_ measured by TZM-bl assay). SHIV-1157ipEL-p is heterologous to HIV1084i Env and SIVmne Gag-Pol immunogens. Group 4, unvaccinated rhesus monkeys (RM) challenged identically with the same virus stock as part of other studies. All RM with no viremia at week 7 (shown in red) were rechallenged i.r. with a single high-dose of SHIV-1157ipEL-p (1.5×10^5^ TCID_50_; red arrows). Group 5: naïve controls for the high-dose challenge. *, Mamu A*001-positive RM. (B) Phylogenetic analysis of Env sequences of immunogen and challenge virus. HIV-1 Env sequences of clade C and non-clade C reference strains were obtained from the Los Alamos HIV-1 sequence database. The evolutionary tree was inferred using the Neighbor-Joining method [47] by MEGA4 software [48]. The immunogen and challenge virus sequences were on different branches within cluster of HIV-C Env sequences.

Two sets of RM were immunized three times with multimeric HIV-C gp160 (from strain HIV1084i), HIV-1 Tat, and either SIVmne-derived (Group 1) or SIVmac239-derived (Group 2) Gag-Pol particles in incomplete Freund's adjuvant (IFA). The controls (Group 3) received IFA alone (Figure 1A). The Env immunogen was 22.1% divergent from Env of the challenge virus; the two Envs were clearly distinct by phylogenetic analysis (Figure 1B). The SIVmne-derived Gag-Pol particles given to Group 1 vaccinees were partially heterologous (5.5% divergent) to Gag-Pol of the challenge virus (Figure S1).

None of the vaccine recipients developed any untoward side effects. No local reactogenicity was noted.

**Figure 2:**
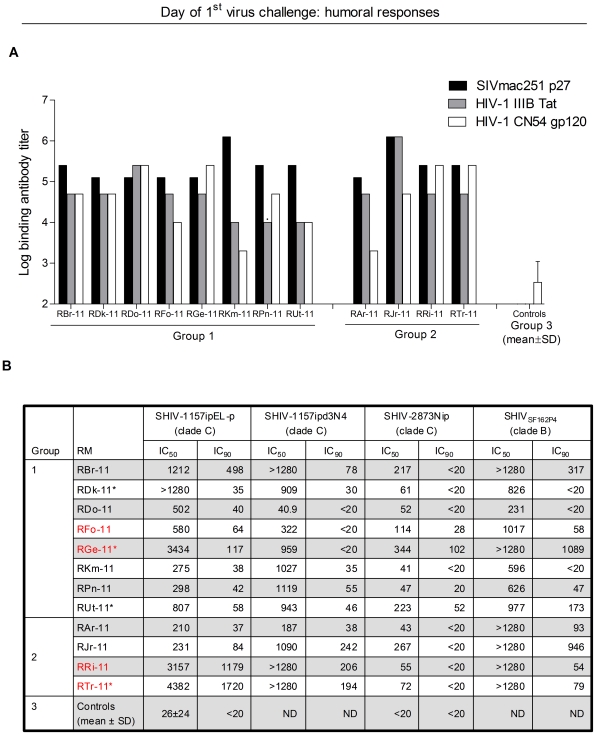
Humoral immunity at the time of initial low-dose SHIV-1157ipEL-p challenge. (A) Reciprocal serum antibody ELISA titers against SIVmac251 p27 Gag, HIV-1 Tat and heterologous clade C HIV_CN54_ gp120. (B) NAb activity SHIV-1157ipEL-p and, SHIV_SF162P4_) and Tier 2 (SHIV-1157ipd3N4, SHIV-2873Nip) viruses was measured in human PBMC in the presence of polymyxin B. TZM-bl neutralization assay was also performed against SHIV-1157ipEL-p. IC_50_ and IC_90_ titers, reciprocal dilutions of serum giving 50% and 90% neutralization (inhibitory concentrations), respectively, are shown. *, Mamu A*001-positive RM.

**Figure 3:**
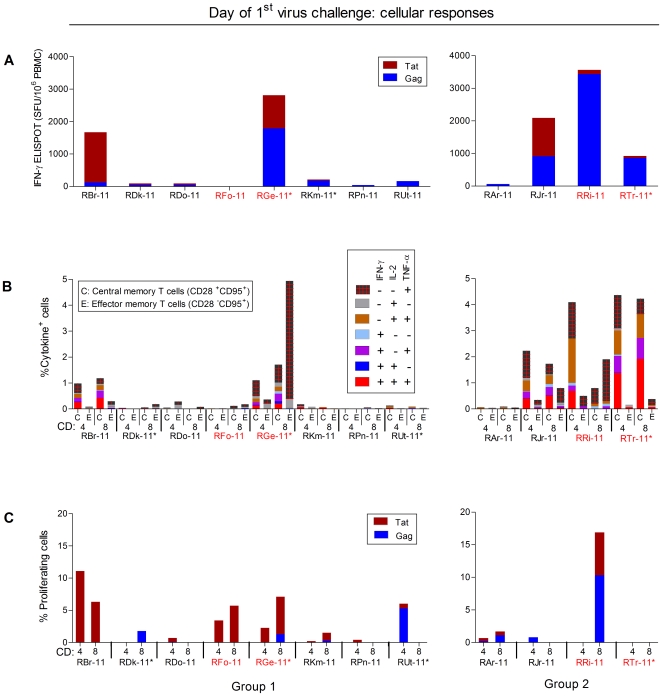
Cellular immunity at the time of initial low-dose SHIV-1157ipEL-p challenge. (A) The frequency of antigen-specific cells measured by IFN-γ ELISPOT assay. No responses were seen in control Group 3. (B) The frequency of SIV Gag and HIV-1 Tat-specific peripheral blood CD4^+^ and CD8^+^ T cells producing intracellular IFN-γ, IL-2 and TNF-α was measured using multiparameter flow cytometry. (C) Proliferation of peripheral blood CD4^+^ and CD8^+^ T cells in response to SIV Gag or HIV-1 Tat proteins. *, Mamu A*001-positive RM.

Virus-specific immune responses measured on the day of challenge (two weeks after last vaccination) are shown in Figures 2 and 3. All vaccinees (Groups 1+2) developed high-titer binding antibodies to SIVmac251 Gag, HIV-1 Tat and HIV-C gp120 (Figure 2A). Results of neutralization assays against various R5 SHIV strains, including heterologous SHIV-1157ipEL-p [12], SHIV-1157ipd3N4 [10], SHIV-2873Nip [11] and SHIV_SF162P4_, a clade B strain [22], are shown in Figure 2B. All 12 vaccinees had nAb responses against the challenge virus, SHIV-1157ipEL-p, by peripheral blood mononuclear cell (PBMC)-based assay.

Two weeks after the last immunization, some vaccinees had strong cellular immune responses to SIV Gag and/or HIV-1 Tat as measured by interferon (IFN)-γ ELISPOT (Figure 3A) and intracellular cytokine staining (ICS) assays (Figure 3B). Three out of 4 RM from Group 2 and one out of 12 RM from Group 1 showed strong ELISPOT- and ICS responses against SIV Gag (Figure 3 A,B). This disparity may be due to the fact that overlapping synthetic peptides used in those assays were of SIVmac239 origin, which is homologous to the Gag-Pol particles given to Group 2 but partially heterologous to those given to Group 1. In some vaccinees (RRi-11, RTr-11, RGe-11), ELISPOT responses were as strong as those induced by highly immunogenic live vectors [23], [24]. SIV Gag-specific responses were seen in both CD4^+^ and CD8^+^ T cells, mostly with central memory phenotypes, and a significant fraction of each cell population displayed polyfunctional cytokine responses (Figure 3B). Most vaccinees also had virus-specific proliferative responses (Figure 3C).

### Protection against mucosal challenges with SHIV-1157ipEL-p

**Figure 4:**
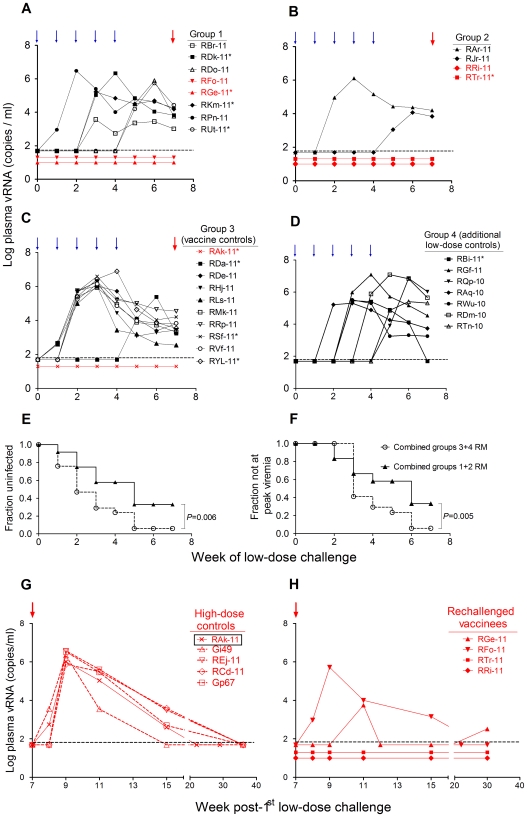
Plasma viral RNA (vRNA) loads after SHIV-1157ipEL-p challenges and Kaplan-Meier plots. (A–D) vRNA loads after low-dose viral challenges for Groups 1-4. Black symbols, vRNA loads of Groups 1-4 during the weekly low-dose challenges (blue arrows); red symbols, vRNA loads of animals later given high-dose challenge (red arrows; see also Figure 1 legend). (E) Kaplan-Meier plots depicting the fraction of monkeys remaining aviremic. (F) Kaplan-Meier plots depicting the fraction of animals not yet having reached peak viremia. (G–H) vRNA loads after a single high-dose SHIV-1157ipEL-p challenge of newly recruited naïve controls (Group 5) plus prior control monkey RAk-11 (G), or rechallenge of vaccinees (H). Probability (*P*) values were determined by 2-sided log-rank analysis. Horizontal dashed lines in A–D, G–H: limit of RT-PCR assay detection (50 vRNA copies/ml; [25]).

All vaccinated (Groups 1 and 2) and control RM (Group 3 plus Group 4, as a part of another study) were given five i.r. low-dose SHIV-1157ipEL-p challenges (8000 50% tissue culture infectious doses (TCID_50_) measured by TZM-bl assay) (Figure 1). Env of the challenge virus was heterologous to the HIV1084i gp160 immunogen (22.1% divergent) (Figure 1B), whereas the challenge virus Gag was partially heterologous to the Gag-Pol particles given to Group 1 (Figure S1). Three weeks after the final low-dose virus exposure, 94% of all controls (Groups 3+4) were infected, compared to 67% of all vaccinees (*P* = 0.07 by Fisher's exact test (one-sided)); monkeys RFo-11, RGe-11, RRi-11 and RTr-11 remained aviremic (Figure 4A–D). The vaccinees' mean peak plasma viral RNA (vRNA) load was significantly lower than the combined controls' (6.6×10^5^ versus 2.8×10^6^ copies/ml; *P* = 0.045, Wilcoxon rank-sum test (for statistical analysis, aviremic RM were assigned a vRNA load of 49 copies/ml, based upon an assay sensitivity of 50 copies/ml [25]). Likewise, the mean 7-week “area under the curve” viral loads differed significantly between the vaccinees and controls (*P* = 0.01). The combined vaccinees of Groups 1+2 also had significant delays in both time until first detectable viremia (Figure 4E) and time until peak viremia (Figure 4F) compared to the combined Groups 3+4 controls. Significant delays of both parameters were also observed when Group 1+2 vaccinees were compared only to Group 3 controls (Figure S2).

Because one control remained virus-negative, all RM without evidence of viremia after five low-dose challenges (shown in red, Figure 1) were rechallenged once i.r. at week 7 with a high dose of SHIV-1157ipEL-p (1.5×10^5^ TCID_50_); these included two monkeys each from Groups 1 and 2 and one from Group 3 (shown in red in Figure 1A). By 2 weeks post-rechallenge, peak vRNA loads of >10^6^ copies/ml plasma were observed in RAk-11 (a control RM of Group 3) plus four identically challenged newly-enrolled naïve RM (Figure 4G). In contrast, two out of the four vaccinees (RRi-11 and RTr-11) remained virus-negative and one, RGe-11, showed only two transient blips of <10^4^ vRNA copies/ml (Figure 4H). No virus was detected ever in RRi-11 and RTr-11 using RT-PCR analysis of peripheral lymph nodes (LN; data not shown) or ultracentrifuged plasma taken at week 4 post-rechallenge (Table S1). Importantly, PBMC from RRi-11, RTr-11 and RGe-11 supported SHIV-1157ipEL-p replication in vitro but only after depletion of CD8^+^ T cells (Figure S3). Thus, the combined data suggest that vaccination completely prevented viremia in two RM and prevented persistent, systemic infection in a third. Three additional monkeys had evidence of viral containment with plasma viral loads ≥1 log lower than the mean plasma vRNA load of all 17 controls of Groups 3+4. Lowering of peak viremia by ≥1 log has been previously used as a measure of relative vaccine-induced viral containment [26], [27]. By this measure, our vaccine strategy provided 50% complete or partial protection.

### Correlates of protection

**Figure 5:**
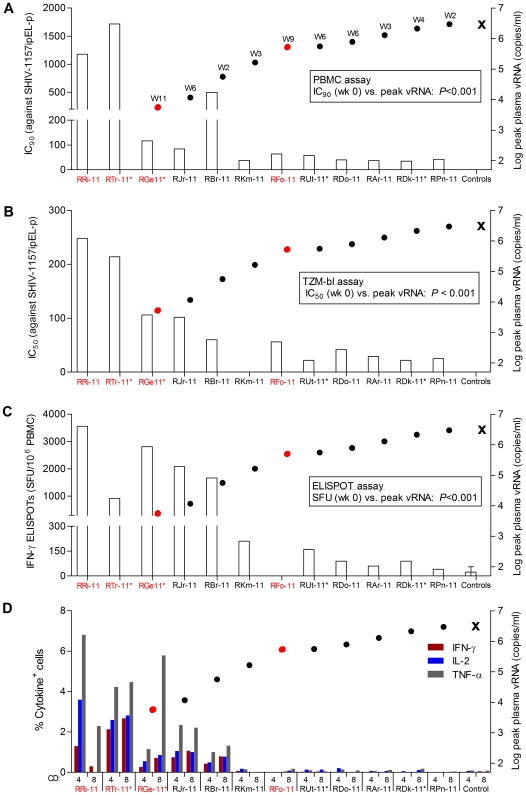
Immune parameters associated with protection. Vaccinees were ranked in ascending order of peak viremia after all virus challenges (animals that received the high-dose challenge are shown in red), and immune parameters measured on the day of 1^st^ low-dose virus challenge are presented. W, week of peak viremia. (A) Neutralizing antibody titer (IC_50_) measured by TZM-bl cell-based assay vs. peak plasma vRNA. (B) Neutralizing antibody titer (IC_90_) measured by PBMC-based assay vs. peak plasma vRNA. (C). SIV Gag- plus HIV-1 Tat-specific IFN-γ ELISPOTs vs. peak plasma vRNA. (D) SIV Gag- plus HIV-1 Tat-specific CD4^+^ and CD8^+^ T cells producing IL-2, IFN-γ and TNF-α vs. peak plasma vRNA. Inverse correlations of peak vRNA with cellular (*P*<0.001) and nAb (*P*<0.001) responses were determined by Spearman analyses. The mean peak plasma viremia of all controls is shown by the X mark. All control animals were tested in neutralization (TZM-bl, PBMC) and ELISPOT assays; one control animal was included in the ICS assays.

Next, we sought to correlate vaccine-induced nAb and/or cellular immune responses, measured at week 0 (day of 1^st^ virus exposure), with viral load parameters following completion of all viral challenges, including high-dose rechallenge. Vaccinees were ranked according to increasing peak vRNA loads (Figure 5); aviremic monkeys (RRi-11 and RTr-11) were assigned vRNA loads of 49 copies/ml (assay sensitivity, 50 copies/ml).

All 12 vaccinees had nAb responses against the challenge virus, SHIV-1157ipEL-p, as measured by PBMC-based assay (Figure 2B and 5A) whereas 11 out of 12 had nAb responses by TZM-bl assay (Figure 5B). NAb titers measured as 90% inhibitory concentration (IC_90_) by PBMC assay as well as 50% inhibitory concentration (IC_50_) measured by TZM-bl assay showed a strong inverse correlation (*P*<0.001) with peak plasma viremia (Figure 5A, 5B). Similarly, cellular immunity, as measured by combined IFN-γ ELISPOT activity against SIV Gag and HIV-1 Tat, also showed a highly significant inverse correlation (*P*<0.001) with peak vRNA loads (Figure 5C). Thus, both humoral and cellular immune responses were significant correlates of protection. To our knowledge, this is the first report that simultaneously linked nAb titers and antiviral cellular immunity with the degree of protection. Furthermore, the cellular immune responses in RM with complete or partial protection were polyfunctional as shown by intracellular production of IFN-γ, IL-2 and TNFα after stimulation with SIV Gag and HIV-1 Tat peptides (Figure 3B and 5D).

**Figure 6:**
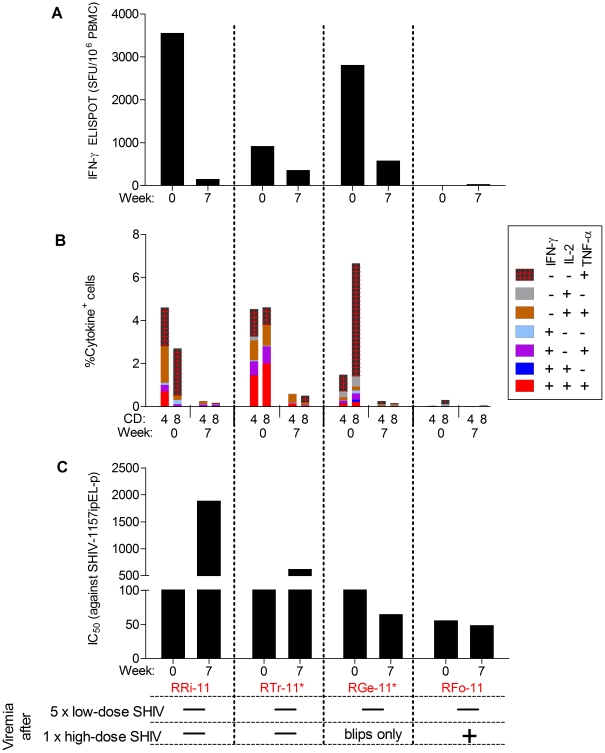
Immune responses on the day of high-dose virus challenge. Cellular and nAb responses at week 0 (day of first virus exposure) and week 7 (day of high-dose virus challenge) for: (A) SIV Gag- and HIV-1 Tat-specific IFN-γ ELISPOT responses. (B) SIV Gag and HIV-1 Tat-specific peripheral blood CD4^+^ and CD8^+^ T cells producing intracellular IFN-γ, IL-2 & TNF-α (measured by multiparameter flow cytometry). (C) nAb titers against the challenge virus (IC_50_ measured by TZM-bl assay).

On the day of high-dose rechallenge (week 7), just prior to virus re-exposure, the SHIV-specific cellular responses had significantly declined compared to week 0 values in three out of the four vaccinees (RRi-11, RTr-11, RGe-11) that had remained aviremic after the initial five low-dose virus exposures (Figure 6A, 6B). We ascribe this lack of anamnestic responses to these monkeys remaining uninfected, either because of an insufficient virus dose or immune protection. The fourth monkey, RFo-11, had no detectable cytokine production at either week 0 or 7 and was infected after the single-high dose exposure.

Next, we assessed nAb responses on the days of 1^st^ low-dose and the one-time high-dose SHIV challenges in the context of the final outcomes for the four vaccinees that had remained aviremic after the five low-dose challenges. While the pattern of nAb titers between weeks 0 and 7 varied, it is important to note that the two RM that remained aviremic after *all* SHIV challenges had nAb titers ≥600 against the challenge virus (measured as IC_50_ by TZM-bl assay) (Figure 6C).

### Assessment of different degrees of protection

Two RM remained completely aviremic despite all virus challenges (RRi-11 and RTr-11). We sought to test whether sterilizing immunity was achieved or whether cryptic infection resulting in viral tissue reservoirs may have occurred despite a lack of measurable plasma viremia at any time point. As mentioned above, post-challenge LN biopsies failed to reveal any viral sequences. While we considered ablating CD8^+^ cells with a cytotoxic monoclonal antibody to allow virus to emerge from tissue reservoirs during a transient loss of virus-specific CD8^+^ T and NK cells, the high nAb levels in the two protected monkeys may likely have prevented viremia. Likewise, the presence of high nAb titers precluded using blood transfer to naïve RM to test for infectious virus. Thus, we chose an alternate strategy to examine the possibility of cryptic infection and our analysis focused on: 1) the generation of new antiviral immune responses against viral targets absent from our vaccine, e.g. Nef, and 2) cryptic infection, as defined by boosting of preexisting cellular antiviral immune responses. The latter implies presentation of viral peptides through MHC class I molecules occurred after at least a few target cells became productively infected.

**Figure 7:**
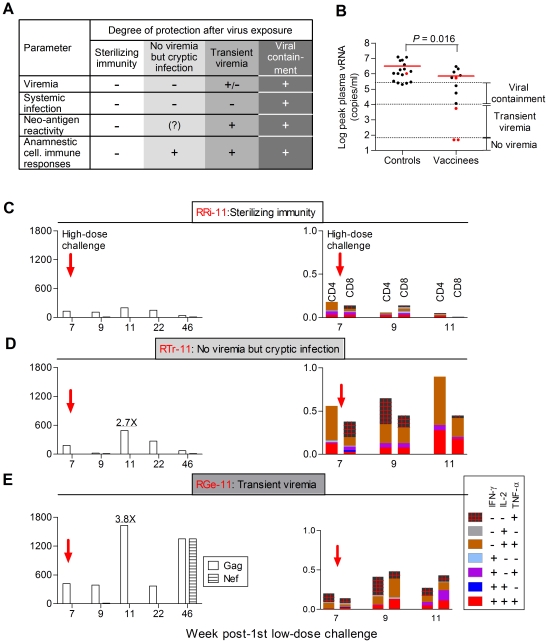
Degrees of protection. (A) Parameters to define different degrees of protection. Transient viremia, vRNA blip(s) (<10^4^ vRNA copies/ml, which is not expected to result in seroconversion); viral containment, lowering of peak vRNA by ≥1 log vs. controls. Anti-Nef (non-vaccine component) responses were measured to detect neo-antigen reactivity. The fold increase in ELISPOTs after high-dose virus challenge compared to the day of high-dose virus challenge was determined to identify anamnestic cellular immune responses. (B) Distribution of peak vRNA. Red lines, means; dashed lines: upper, viremia 1 log lower than mean of controls; middle, 1×10^4^ vRNA copies/ml; lower, limit of vRNA detection (50 copies/ml). (C–E) Anamnestic cellular responses and neo-antigen reactivity in the monkeys indicated. Left panels, IFN-γ ELISPOTs against SIV Gag (open bars) and Nef (striped bars, neo-antigen). Right panels, SIV Gag-specific intracellular cytokine staining.

Parameters defining different degrees of protection following viral exposures are summarized in Figure 7A. The distribution of peak vRNA loads for all controls and vaccinees along with a schema of the different protection levels are shown in Figure 7B. Remarkably, monkey RRi-11 fulfilled all criteria for sterilizing immunity (Figure 7C). Cellular immunity measured by SIV Gag- and HIV-1 Tat-specific ELISPOTs were very high after vaccination (>3,500 combined SFU/10^6^ PBMC). At the time of high-dose rechallenge, these responses had dropped to <200 SFU/10^6^ PBMC and remained low even after rechallenge (Figure 7C, left panel). ICS also showed no anamnestic cellular responses (Figure 7C, right panel). In contrast, monkey RTr-11 had a clear anamnestic cellular immune response at 4 weeks post-high-dose virus challenge (week 11; Figure 7D), even though the RM was never viremic and no viral sequences were found in LN tissue. A third animal, RGe-11, only had two low-level blips of viremia, but negative LN biopsies. Anamnestic cellular immune responses were clearly seen at week 11 in this RM (Figure 7E), which also mounted novel cellular responses against Nef.

## Discussion

After all virus challenges, we achieved 50% protection overall, combining cases of partial protection (i.e., ≥1 log lower peak viremia compared to the controls, the same parameter used in earlier studies [26], [27]), protection from persistent systemic viremia (i.e., only transient viremia or cryptic infection), and sterilizing immunity. Our data give proof-of-concept that a high degree of protection can be achieved with a vaccine strategy designed to recruit cellular as well as humoral immune defenses. To our knowledge, vaccine protection against a challenge virus carrying a completely mismatched HIV-C envelope has been linked for the first time to the simultaneous induction of cellular as well as nAb responses with a high degree of significance.

The two envelopes used to generate either the gp160 immunogen or the challenge R5 clade C SHIV were derived from distinct HIV-C strains (HIV1084i and HIV1157i, respectively), and the 22% amino acid divergence between the two envelopes is representative of intra-community sequence variability since both HIV-C strains were isolated from infected infants in Lusaka, Zambia [12], [21]. Thus, our model was as true-to-life as possible for approximating HIV-1 exposure among humans exposed to locally circulating virus strains.

Our multiple low-dose, mucosal R5 SHIV challenge model was also designed to better reflect the relatively low infectious inocula, which are thought to be involved during mucosal HIV-1 exposure of humans [28], rather than the single high-dose challenges used in earlier primate studies. The control animal (RAk-11) that remained aviremic during the low-dose challenge phase can be considered as representative of an uneventful mucosal virus exposure. The same can be said about the vaccinee RFo-11 that had remained virus-free after the low-dose challenges but promptly became highly viremic after high-dose challenge. This RM had failed to mount significant ELISPOT and ICS responses and had only relatively low nAb titers after the completion of the vaccine regimen. Our experimental design allowed a separate statistical analysis of the low-dose challenge phase while the subsequent one-time high-dose challenge permitted us to differentiate between insufficient virus challenge dose and vaccine effects. Overall, we observed substantial vaccine efficacy during the low-dose challenge phase. Out of 17 control RM, 16 became viremic (94%), in contrast to a 67% infection rate among all 12 vaccinees. The vaccinees had significant delays in first-detectable viremia, lower peak vRNA loads and area-under-the curve values compared to the controls.

To our knowledge, this is the first study to test the immunogenicity and efficacy of multimeric gp160 from a recently transmitted HIV-C against multiple low-dose challenges with an R5 SHIV that also encoded a recently transmitted envelope from a distinct viral isolate. Emerging data indicate that the configuration of the HIV-C envelope from recently transmitted viruses differs significantly from late-stage HIV-C Envs [29], [30]. In the context of HIV-1 infection, clade C Envs are also more immunogenic and more likely to induce cross-neutralizing Abs than HIV-1 clade B Envs [31]. Our induction of strong nAb responses against heterologous SHIV-C (SHIV-1157ipEL-p) and detectable neutralization of heterologous viruses (SHIV-1157ipd3N4 and SHIV-2873Nip) warrant the use of Env immunogens derived from recently transmitted HIV-C strains.

Oligomeric gp140 or gp160 immunogens have been tested in macaques; generally, nAb breadth was somewhat limited [32]. Immunization of macaques with V2-deleted SF162 gp140 trimers induced nAbs and protected 12 out of 12 monkeys against mucosal challenge with SHIV_SF162P4_ [33]; however, the challenge virus exactly matched the immunogen and thus is not reflective of a real-life situation, in which human AIDS vaccine recipients will be exposed to HIV-1 strains that do not match their vaccine. Here we report protection against a challenge virus with 22.1% Env divergence from the immunogen.

We performed our immunizations by the intramuscular (i.m) route. For HIV-1 Env protein immunization, the i.m route was described as more immunogenic compared to the mucosal route [33]. In that study, i.m. immunized monkeys were protected against homologous clade B SHIV challenge but not those immunized only mucosally.

Non-human primate model studies involving R5 SHIV challenges may be used to obtain information about a critical parameter in AIDS vaccine development: the nAb titer that needs to be achieved to protect against systemic infection. The issue of which nAb assay to employ for immunogenicity screening is currently under intense study. Our data show that nAb titers vary significantly depending on the assay system used, including target cells. The PBMC assay-based IC_90_ values for monkeys RRi-11 and RTr-11 (vaccinees that never showed viremia) on the day of first virus exposure were >1000. In contrast, the TZM-bl assay yielded IC_50_ values in the range of 200 to 300. The fact that TZM-bl assay-derived nAb titers were significantly lower is in agreement with findings of others [34], [35]. Nonetheless, the overall rank order of anti-SHIV-1157ipEL-p nAb responses measured by the two assays followed a similar although not identical pattern and both assays ranked RRi-11 and RTr-11 at the top among all vaccinees.

Although our functional assays of humoral immunity focused on neutralizing activity, other antibody effector functions could have contributed to the overall vaccine efficacy in our study, such as antibody-dependent cellular cytotoxicity (ADCC) and antibody-dependent cell-mediated viral inhibition (ADCVI) [36], [37]. Analysis of such additional antibody effector functions in our study is complicated by the very high challenge virus-specific neutralizing activity shown by the protected vaccinees.

A recent study reported antibody-mediated transcytosis inhibition of SHIV89.6P across the epithelial layer [38] with non-neutralizing polyclonal antibodies. Inhibition of transcytosis in the complete absence of standard neutralizing activity was described as the protective mechanism [39]. Lastly, multimeric IgM and IgA antibodies can aggregate virions and inhibit movement through mucosa and epithelial layers [40]. Inhibition of virus penetration through mucosal barriers may be achieved by such antiviral antibody functions that differ from standard neutralization and may also be beneficial.

Recombinant protein immunization induced both humoral and cellular immunity that included both CD4 as well as CD8 T-cell responses and is in agreement with data of others [41]. We also noted that the protected RM in our study mounted virus-specific polyfunctional cellular immune responses while such responses were either low or undetectable among unprotected vaccinees. These results support the notion that polyfunctional T cells can provide a better degree of protection [42], [43].

It has been widely discussed that for protection against mucosal virus challenges, immune responses in mucosal compartments are highly desired. However, we had to refrain from performing rectal biopsies in order to maintain mucosal integrity during the multiple low-dose SHIV-C challenges.

During the follow-up time of our study, post-acute viremia did not differ between infected vaccinees and controls. Interestingly, this parallels the results of the RV144 trial, as there was no significant difference in mean viral loads between vaccinees with HIV-1 infection compared to infected individuals in the placebo group [3]. Remarkably, in our primate study, one third of all vaccinees remained aviremic throughout the initial low-dose challenge phase. While these data are encouraging, the task will be to improve the potency of the cellular immunity against heterologous viruses and to develop immunogens capable of generating nAb responses with increased breadth to not only neutralize viruses circulating within the same local community, but also more divergent HIV-C isolates as well as HIV-1 strains of different clades. Our study provides evidence for the importance of generating balanced cellular and humoral immune responses by candidate AIDS vaccines.

## Methods

### Animals

This study was carried out in strict accordance with the recommendations in the Guide for the Care and Use of Laboratory Animals of the U.S. Public Health Services/National Institutes of Health, as well as according to the recommendations in the Weatherall report on “The Use of Non-human Primates in Research” (http://www.acmedsci.ac.uk/images/project/nhpdownl.pdf). The protocol was approved by the Committee on the Ethics of Animal Experiments of Emory University (IACUC ID: 261-2008Y; Emory University Animal Welfare Assurance Number A3180-01). The rhesus monkeys were housed at the Yerkes National Primate Research Center (YNPRC, Emory University, Atlanta, GA). YNPRC facilities are fully accredited by the Association for Assessment and Accreditation of Laboratory Animal Care International. Animal experiments were approved by the Institutional Animal Care and Use Committees at Emory and the Dana-Farber Cancer Institute. All animal procedures were performed under ketamine-telazol anesthesia. Because the experiments described here involved a virus that may cause an incurable disease, such as AIDS, discomfort, stress and pain may occur. Animals were closely monitored and observed for development of disease at least twice daily. If the animals are determined to be under stress or in discomfort, appropriate anesthetics and/or analgesics are administered as directed by the clinical veterinary staff. Euthanasia is also an option should treatment not alleviate stress. In the current study, no untoward clinical problems were noted, and none of the virus-infected monkeys progressed to AIDS.

All animals were Mamu B*008 and B*017 negative; Mamu A*001^+^ RM are indicated in all Figures by an asterisk (*).

### Viruses

HIV1084i [21] was isolated from a Zambian infant and was used for the preparation of multimeric gp160 immunogen. The challenge virus, SHIV-1157ipEL-p, was generated as described earlier [12]. An animal-titrated stock of SHIV-1157ipEL-p was prepared using Con A-stimulated RM PBMC cultured in the presence of TNF-α (10 ng/ml). For low- and high-dose SHIV-1157ipEL-p challenges, inocula of 8,000 TCID_50_ and 1.5×10^5^ TCID_50_ (titrated on TZM-bl cells) were used, respectively.

### Immunogens

HIV-1 Tat was purchased from Advanced Bioscience Laboratories, Inc. (Kensington, MD). SIVmac239 Gag-Pol particles, SIVmne Gag-Pol particles and multimeric HIV1084i gp160 were produced from recombinant vaccinia virus-infected BSC-40 cells [44], [45]. For each immunization, 100 µg of each protein in incomplete Freund's adjuvant (IFA) was administered i.m.

### Measurement of plasma vRNA

Plasma vRNA was isolated by QiaAmp Viral RNA Mini-Kit (Qiagen, Germantown, MD); vRNA levels were measured by quantitative reverse-transcriptase polymerase chain reaction (RT-PCR) for SIV *gag* sequences [25]. Additionally, primers/probes according to Lifson were used [46]. Assay sensitivity was 50 vRNA copies/ml [25].

### Interferon-γ ELISPOT assay

Multiscreen-IP plates (Millipore, Billerica, MA) were coated with anti-human IFN-γ antibody (clone B27, BD Pharmingen, San Jose, CA), blocked with 10% heat inactivated fetal bovine serum (FBS) in RPMI-1640 (R-10). 1×10^5^ cells were incubated overnight with SIVmac239 Gag or HIV-1 consensus B Tat peptides (each peptide, 2 µg/ml) obtained through NIH AIDS Research and Reference Reagent Program (ARRRP). The IFN-γ-secreting cells were detected by using biotinylated anti-human IFN-γ antibody (clone 7-B6-1, Mabtech, Cincinnati, OH), Horseradish peroxidase-conjugated streptavidin (BD Biosciences, San Jose, CA) and AEC chromogen substrate (BD Biosciences). The spots were enumerated using an Immunospot ELISPOT reader (CTL, Cleveland, OH). Assays were done in duplicates and background counts with no peptide stimulation were subtracted.

### Intracellular cytokine staining

PBMC were suspended in R-10 containing Brefeldin A (10 µg/ml; Sigma-Aldrich, St. Louis, MO), Monensin (Golgistop, BD Biosciences), anti-CD49d antibody (clone 9F10, BD Biosciences) and anti-CD28-PECy7 antibody (clone CD28.2, eBioscience, San Diego, CA). The cells were stimulated with SIVmac239 Gag or HIV-1 Tat peptides at 37°C for 6 h. Cells were stained with anti-CD3-AlexaFluor700 (clone SP34-2, BD-Pharmingen), anti-CD4-PE (clone M-T477, BD-Pharmingen), anti-CD8-AmCyan (clone SK1, BD-Biosciences) and anti-CD95-PE-Cy5 (clone DX2, BioLegend, San Diego, CA) antibodies. The cells were then fixed/permeabilized with Cytofix/Cytoperm (BD Pharmingen) and stained intracellularly with anti-IL-2-APC (clone MQ1-17H12, BD Pharmingen), anti-IFN-γ-PerCP/Cy5.5 (clone 4S.B3, BioLegend) and anti-TNF-α-PacificBlue (clone MAb11, eBioscience) antibodies. At least 50,000 lymphocytes were acquired on LSR-II, BD Immunocytometry Systems and data was analyzed using FlowJo 6.0 (Tree Star, Inc., Ashland, OR) software. Percentages of CD3^+^CD4^+^ and CD3^+^CD8^+^ cells producing each of seven different possible combinations of IL-2, IFN-γ and TNF-α were determined. Background numbers of cells producing cytokines without peptide stimulation were subtracted.

### Lymphocyte proliferation assay

PBMC were stained with CFSE (CellTrace™ CFSE Cell Proliferation Kit, Invitrogen, Camarillo, CA) and incubated with or without antigen (SIV Gag, HIV-1 Tat; 2 µg/ml) for 5 days at 37°C. After incubation cells were stained with anti-CD3-Alexa Fluor 700 (clone SP34-2), anti-CD4-PerCP (clone L200) and anti-CD8-PE (clone RPA-T8) antibodies. The percentage of proliferating CD3^+^CD4^+^ and CD3^+^CD8^+^ cells was determined using FACSDiva (BD Biosciences) software. Background proliferation without any stimuli was subtracted.

### Serum antibody binding titers

ELISA plates were coated with SIVmac251 p27 (Immunodiagnostics, Inc., Woburn, MA), HIV-1 Tat and HIV_CN54_ gp120 (ARRRP), diluted to 2 µg/ml. After blocking with 3% BSA, serial serum dilutions were added in duplicate wells. Antibody binding was detected by horseradish peroxidase conjugated rabbit anti-monkey IgG (Sigma-Aldrich, St. Louis, MO) and O-Phenylenediamine dihydrochloride (OPD) + hydrogen peroxide substrate. Titers were calculated as the greatest reciprocal serum dilution giving OD readings >mean + 5 standard deviations above background as measured using preimmune serum from the same RM at the same dilution.

The HIV-C_CN54_ gp120 produced in insect cells was used to measure anti-Env binding antibody titers. This ruled out the possibility of detection of anti-vaccinia antibodies that might have been induced due to vaccinia proteins possibly associated with our immunogens that were produced by recombinant vaccinia technology.

### PBMC-based neutralization assay

Sera were tested against SHIV-1157ipEL-p, SHIV-2873Nip and SHIV_SF162P4_ in PBMC-based neutralization assays, considered the gold-standard currently [35]. Predetermined virus inocula were incubated with diluted sera in triplicate wells at 37°C for 1 h. Virus was also incubated with medium alone (to determine 100% virus production). After incubation, 5×10^5^ phytohemaglutinin (PHA)-stimulated human PBMC were added to all wells in the presence of IL-2 (40 U/ml) and polymyxin B (15 µg/ml). From day 3 to 8, 100 µl/well of supernatant was collected and replaced with medium. Supernatants were tested for SIV p27 by ELISA (Advanced Bioscience Laboratories, Kensington, MD). The % neutralization was determined for each serum sample as: %neutralization = 100−[(avg. p27 in wells containing virus+plasma mixture×100)/avg p27 in wells assigned for virus production]. Pooled sera collected from naïve RM were used as negative control, and a mixture of four broadly reactive nAbs (b12, 2F5, 4E10 and 2G12) served as positive control. Serum dilutions that showed 50 and 90% inhibition of virus replication were determined by logarithmic regression analysis.

### TZM-bl cell-based neutralization assay

Sera collected from all monkeys on the day of the first low-dose virus challenge were tested to determine nAb titers against the challenge virus (SHIV-1157ipEL-p). Human PBMC-grown virus was incubated with different serum dilutions and virus neutralization was determined by using the TZM-bl cell-based assay as described [30].

### Statistical analysis

Log-rank comparisons were used for time-to-infection and time-to-peak viremia analyses between vaccinees vs. controls. Wilcoxon rank sum test was used to compare continuous variables (peak viremia and area-under-the-curve) between groups. Associations between continuous variables (peak viremia and challenge virus-specific immune responses, i.e. total ELISPOTs and IC_90_ titers) were assessed using Spearman correlation analysis. Comparison of the number of animals infected between vaccinees vs. controls was assessed using the Fisher's exact test. The reported *P*-values are based on two-sided testing, unless specified otherwise.

## Supporting Information

Figure S1 Phylogenetic analysis of Gag sequences of immunogen and challenge virus. The Gag sequences of SIV and reference HIV strains were obtained from Los Alamos HIV sequence database. The evolutionary tree was inferred using the Neighbor-Joining method by MEGA4 software. The immunogen and challenge virus sequences are labeled with filled black circle.
(TIF)

Figure S2 All vaccinated (Group 1+ 2 RM) animals vs. Group 3 (control) RM. Kaplan-Meier plots depicting the fraction of RM remaining aviremic (A) or not yet having reached peak viremia (B) after low-dose SHIV-1157ipEL-p challenges. *P* values were determined by 2-sided log rank analysis.
(TIF)

Figure S3 In vitro SHIV-1157ipEL-p replication in the PBMC of protected animals. PBMC of the protected animals (RRi-11, RTr-11, RGe-11), collected 4 weeks after high-dose virus challenge were stimulated with concanavalin A (5 µg/ml) in the presence of IL-2 (10 U/ml); 2×10^6^ unfractionated PBMC (A) or CD8^+^ cell-depleted PBMC (B) were exposed to SHIV-1157ipEL-p (1×10^4^ TCID_50_). Virus replication was monitored by p27 ELISA of culture supernatants. As control, virus replication in PBMC of a naïve RM was also measured. Suppression of ex-vivo HIV-1 replication in cultured CD4^+^ T cells by autologous CD8^+^ T cells from HIV-1-infected non-progressors has been reported [49]. Similarly, inhibition of ex-vivo SIV replication in cultured macrophages by MHC- matched Gag- and Nef-specific CD4^+^ T cells from SIV-infected rhesus macaques has been reported [50].
(TIF)

Table S1 Viral RNA copies before and after ultracentrifugation. Ten ml of plasma from monkeys RRi-11, RTr-11, and RGe-11 collected 4 weeks after high-dose SHIV-1157ipEL-p rechallenge were ultracentrifuged (140,000×g for 5 h at 4°C) and the pellets resuspended in 150 µl of PBS. ^1^RFa-10 was a chronically infected RM with SHIV-1157ipEL-p from another study.
(PPT)
