# Supplementary figures and images for: Vaccination against Heterologous R5 Clade C SHIV: Prevention of Infection and Correlates of Protection
Source: PLoS One. 2011 Jul 20;6(7):e22010. doi: 10.1371/journal.pone.0022010 (PMC3140488; doi:10.1371/journal.pone.0022010)

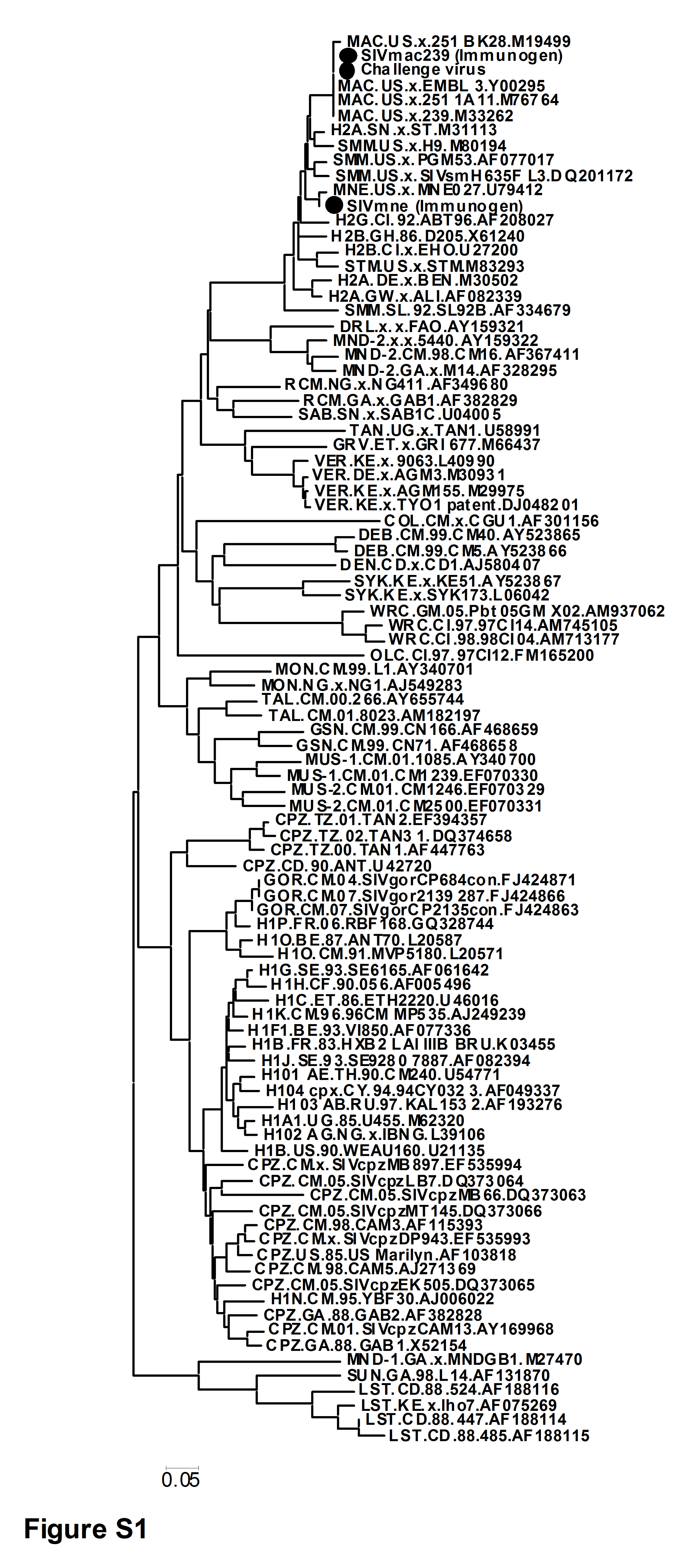

Supplement: Figure S1 — Phylogenetic analysis of Gag sequences of immunogen and challenge virus. The Gag sequences of SIV and reference HIV strains were obtained from Los Alamos HIV sequence database. The evolutionary tree was inferred using the Neighbor-Joining method by MEGA4 software. The immunogen and challenge virus sequences are labeled with filled black circle. (TIF) [file pone.0022010.s001.tif]

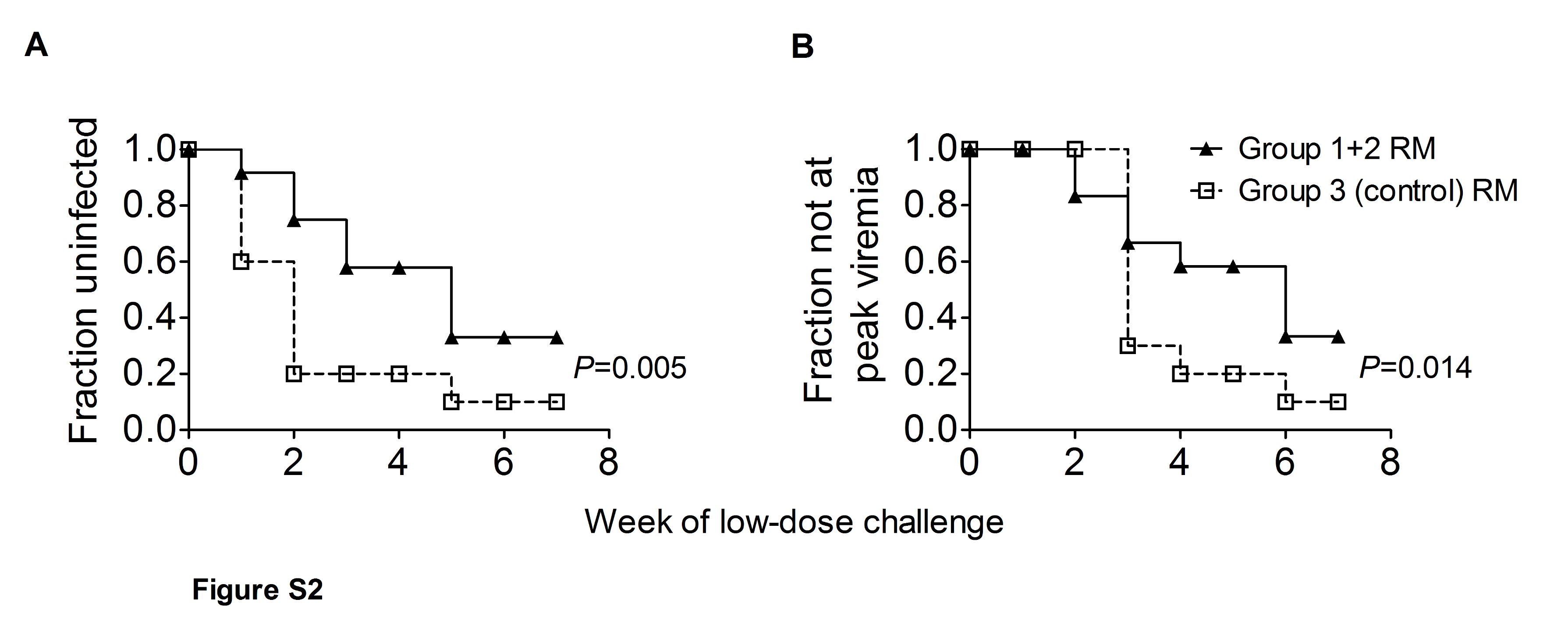

Supplement: Figure S2 — All vaccinated (Group 1+ 2 RM) animals vs. Group 3 (control) RM. Kaplan-Meier plots depicting the fraction of RM remaining aviremic (A) or not yet having reached peak viremia (B) after low-dose SHIV-1157ipEL-p challenges. P values were determined by 2-sided log rank analysis. (TIF) [file pone.0022010.s002.tif]

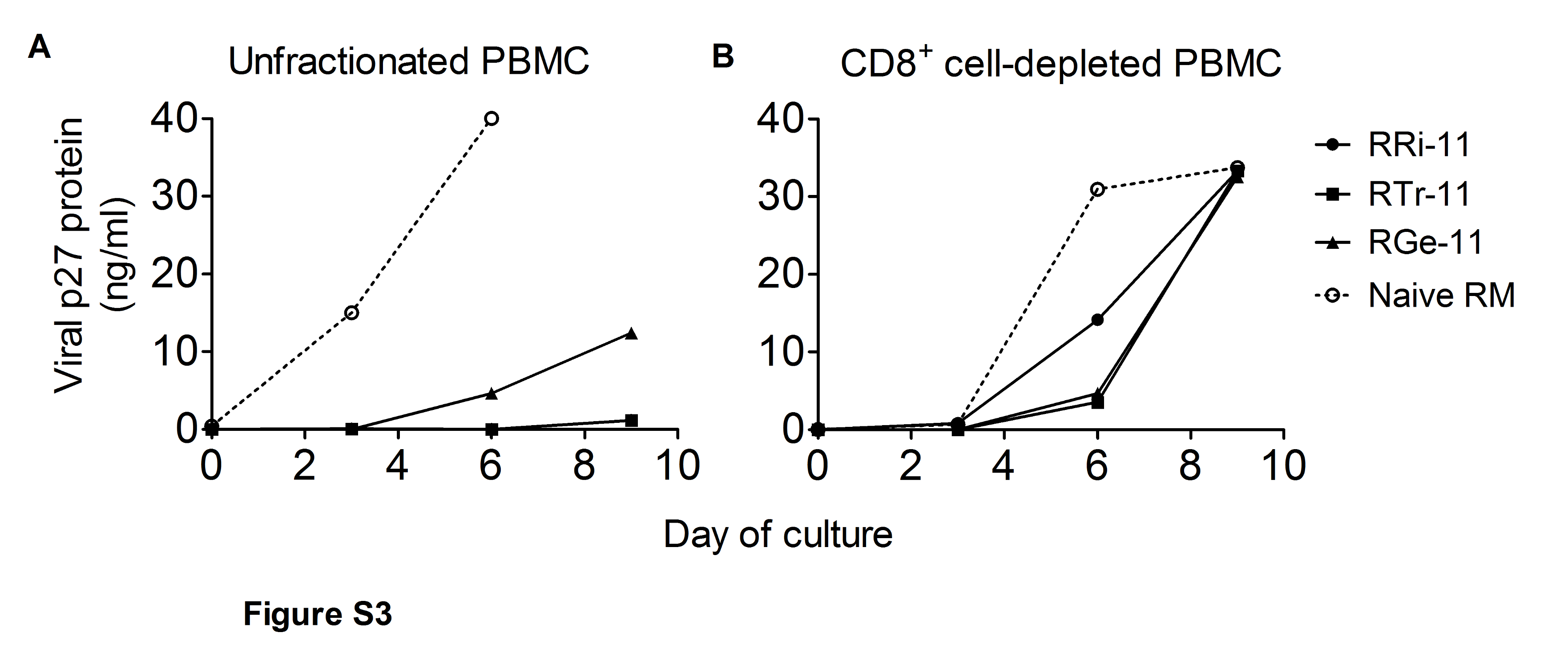

Supplement: Figure S3 — In vitro SHIV-1157ipEL-p replication in the PBMC of protected animals. PBMC of the protected animals (RRi-11, RTr-11, RGe-11), collected 4 weeks after high-dose virus challenge were stimulated with concanavalin A (5 µg/ml) in the presence of IL-2 (10 U/ml); 2×10^6^ unfractionated PBMC (A) or CD8+ cell-depleted PBMC (B) were exposed to SHIV-1157ipEL-p (1×10^4^ TCID50). Virus replication was monitored by p27 ELISA of culture supernatants. As control, virus replication in PBMC of a naïve RM was also measured. Suppression of ex-vivo HIV-1 replication in cultured CD4+ T cells by autologous CD8+ T cells from HIV-1-infected non-progressors has been reported [49]. Similarly, inhibition of ex-vivo SIV replication in cultured macrophages by MHC- matched Gag- and Nef-specific CD4+ T cells from SIV-infected rhesus macaques has been reported [50]. (TIF) [file pone.0022010.s003.tif]
