# Supplementary material for: Vaccination against Heterologous R5 Clade C SHIV: Prevention of Infection and Correlates of Protection
Source: PLoS One. 2011 Jul 20;6(7):e22010. doi: 10.1371/journal.pone.0022010 (PMC3140488; doi:10.1371/journal.pone.0022010)
Supplement: Table S1 — Viral RNA copies before and after ultracentrifugation. Ten ml of plasma from monkeys RRi-11, RTr-11, and RGe-11 collected 4 weeks after high-dose SHIV-1157ipEL-p rechallenge were ultracentrifuged (140,000×g for 5 h at 4°C) and the pellets resuspended in 150 µl of PBS. 1RFa-10 was a chronically infected RM with SHIV-1157ipEL-p from another study. (PPT) [file pone.0022010.s004.ppt]

## Slide 1
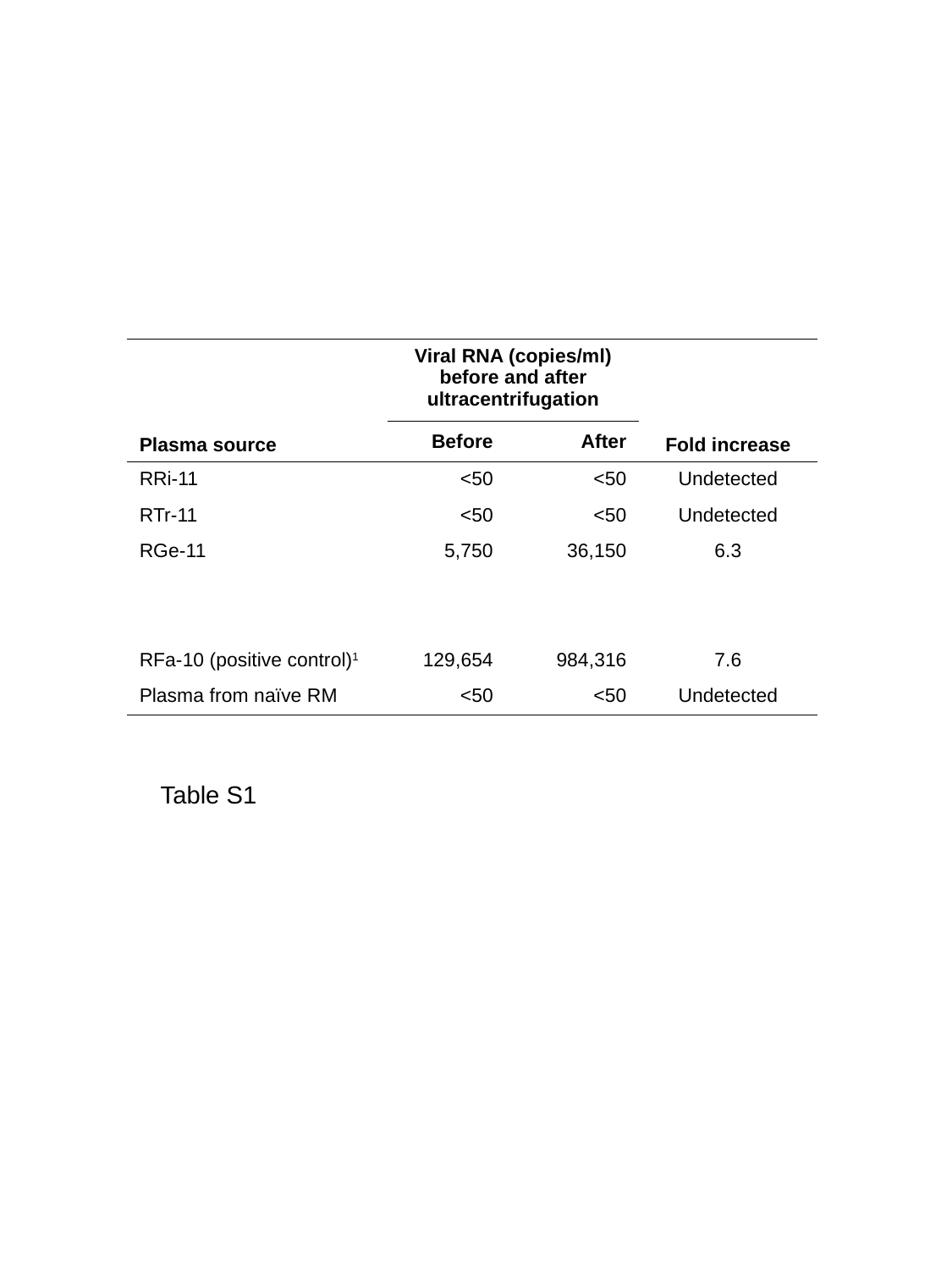

| Plasma source | Viral RNA (copies/ml) before and after ultracentrifugation | | Fold increase |
| --- | --- | --- | --- |
| | Before | After | |
| RRi-11 | <50 | <50 | Undetected |
| RTr-11 | <50 | <50 | Undetected |
| RGe-11 | 5,750 | 36,150 | 6.3 |
| | | | |
| RFa-10 (positive control)1 | 129,654 | 984,316 | 7.6 |
| Plasma from naïve RM | <50 | <50 | Undetected |
Table S1
